# A reduction in Drp1-mediated fission compromises mitochondrial health in autosomal recessive spastic ataxia of Charlevoix Saguenay

**DOI:** 10.1093/hmg/ddw173

**Published:** 2016-06-10

**Authors:** Teisha Y. Bradshaw, Lisa E.L. Romano, Emma J. Duncan, Suran Nethisinghe, Rosella Abeti, Gregory J. Michael, Paola Giunti, Sascha Vermeer, J. Paul Chapple

**Affiliations:** 1 William Harvey Research Institute, Barts and the London School of Medicine, Queen Mary University of London, London EC1M 6BQ, United Kingdom; 2 Blizard Institute, Barts and the London School of Medicine and Dentistry, Queen Mary University of London, London E1 2AT, United Kingdom; 3 Department of Molecular Neuroscience, UCL Institute of Neurology, London WC1N 3BG, United Kingdom; 4 Department of Clinical Genetics, The Netherlands Cancer Institute, 1066 CX Amsterdam, The Netherlands

## Abstract

The neurodegenerative disease autosomal recessive spastic ataxia of Charlevoix Saguenay (ARSACS) is caused by loss of function of sacsin, a modular protein that is required for normal mitochondrial network organization. To further understand cellular consequences of loss of sacsin, we performed microarray analyses in sacsin knockdown cells and ARSACS patient fibroblasts. This identified altered transcript levels for oxidative phosphorylation and oxidative stress genes. These changes in mitochondrial gene networks were validated by quantitative reverse transcription PCR. Functional impairment of oxidative phosphorylation was then demonstrated by comparison of mitochondria bioenergetics through extracellular flux analyses. Moreover, staining with the mitochondrial-specific fluorescent probe MitoSox suggested increased levels of superoxide in patient cells with reduced levels of sacsin.

Key to maintaining mitochondrial health is mitochondrial fission, which facilitates the dynamic exchange of mitochondrial components and separates damaged parts of the mitochondrial network for selective elimination by mitophagy. Fission is dependent on dynamin-related protein 1 (Drp1), which is recruited to prospective sites of division where it mediates scission. In sacsin knockdown cells and ARSACS fibroblasts, we observed a decreased incidence of mitochondrial associated Drp1 foci. This phenotype persists even when fission is induced by drug treatment. Mitochondrial-associated Drp1 foci are also smaller in sacsin knockdown cells and ARSACS fibroblasts. These data suggest a model for ARSACS where neurons with reduced levels of sacsin are compromised in their ability to recruit or retain Drp1 at the mitochondrial membrane leading to a decline in mitochondrial health, potentially through impaired mitochondrial quality control.

## Introduction

The neurodegenerative disease autosomal recessive spastic ataxia of Charlevoix Saguenay (ARSACS: OMIM 270550) was originally identified in Quebec, but is now known to have a worldwide distribution (1,2). ARSACS is regarded as the second most common recessive ataxia and typically presents with a prominent cerebellar ataxia, spasticity and peripheral neuropathy. Central to ARSACS pathology is Purkinje cell loss, which has been documented from patient brain and observed in a mouse model of the disease (3–5).

ARSACS is caused by mutations in the *SACS* gene that encodes sacsin/DNAJC29 (1). At 4579 amino acids in length, sacsin is one of the largest proteins encoded by the human genome. It is a modular protein that from N to C terminus is composed of a ubiquitin-like (UBL) domain that binds to the proteasome (6), three large sacsin repeat regions (SRR) that may have an Hsp90-like chaperone function (7), a J-domain that binds Hsp70 (6,8) and a higher eukaryotes and prokaryotes nucleotide-binding domain that can dimerize (6–10). Despite the identification of these domains, sacsin’s cellular role is unknown.

We have previously reported that siRNA-mediated knockdown of sacsin leads to a reduction in mitochondrial membrane potential and a more interconnected mitochondrial network in the SH-SY5Y neuroblastoma cell line (3). Mitochondrial membrane potential was also reduced in Purkinje neurons from sacsin knockout mice (3). Subsequently, it has been shown that mitochondria are elongated and have reduced mobility in motor neuron cultures from these KO mice (4), whereas in cultured rat hippocampal neurons transduced with lentivirus encoding shRNAmiRs targeting sacsin, mitochondria are clustered and accumulate in the soma and proximal dendrites (3).

There is increasing evidence that perturbation of the equilibrium between mitochondrial fission and fusion underlies mitochondrial defects that contribute to age-related neurodegenerative diseases, including Alzheimer's and Parkinson's (11,12). This is because mitochondrial dynamics are essential for distribution, traffic, and quality control of mitochondria. For mitochondrial quality control, fission is required to sequester damaged parts of mitochondrial networks away from healthy mitochondria, acting as a precursor to their degradation by mitophagy (13). Importantly, deficits in mitochondrial fission may be particularly detrimental in neurons as they have bioenergetic demands at sites distant from the cell body requiring that they generate mitochondria of the correct size for transport along dendrites and axons. There is also the possibility that mitochondrial turnover may be restricted to the cell body, as opposed to occurring in axons (14), further linking mitochondrial dynamics and traffic to mitophagy. Moreover, mitochondrial dynamics facilitates the exchange of mitochondrial matrix and membrane components between individual mitochondrion as a part of mitochondrial network maintenance.

As mitochondrial quality control mechanisms are intrinsically linked to mitochondrial division, disruption of fission has potential downstream consequences for important aspects of mitochondrial function. These include impaired oxidative phosphorylation. Unhealthy mitochondria are also likely to accumulate reactive oxygen species (ROS) and other markers of damage. Moreover, there is evidence of reduced mitochondrial fitness in multiple neurodegenerative diseases. For example, in Parkinson’s, a disease where the mitochondrial quality control machinery is compromised, respiration is impaired while ROS and antioxidant enzymes increase (15,16). Evidence supporting impaired bioenergetics in Parkinson’s includes that fibroblasts from patients with sporadic disease show a significant decrease in respiration compared with controls (17).

The mechanism through which loss of sacsin impairs mitochondrial function, distribution, and network organization is unclear, but could potentially be explained by either an increase in mitochondrial fusion or a decrease in fission. Supporting a model where loss of sacsin impairs fission we identified, through immunoprecipitation, an interaction between the N-terminal 1368 amino acids of sacsin (encompassing the UBL domain and first SRR) and the dynamin-related GTPase dynamin-related protein 1 (Drp1) (3). During mitochondrial fission Drp1 is recruited from the cytosol onto the outer mitochondrial membrane (OMM), where it assembles into foci. These foci consist of oligomeric Drp1 complexes that are thought to wrap around and constrict the mitochondrial tubule to mediate fission (18).

In this study, we used unbiased microarray and gene ontology analyses to identify oxidative phosphorylation and oxidative stress pathways as altered in sacsin knockdown cells and ARSACS patient fibroblasts. Changes in transcript levels for genes in these pathways were confirmed by quantitative RT-PCR (RT-qPCR) in both sacsin knockdown cells and ARSACS patient fibroblasts. Bioenergetics profiles for cells lacking sacsin and accumulation of ROS were also analyzed. Using siRNA-mediated knockdown and ARSACS patient fibroblast cell lines, we also examined the effect of reduced cellular levels of sacsin on Drp1 mitochondrial localization. We identified a reduction in Drp1 foci at mitochondria and reduced translocation of Drp1 to mitochondria after ablation of mitochondrial membrane potential. This suggests that sacsin may play a role in recruitment or retention of Drp1 at mitochondrial fission sites, with loss of this function leading to impaired mitochondrial health.

## Results

### Microarray analyses implicate altered mitochondrial function in ARSACS

**Figure 1.**
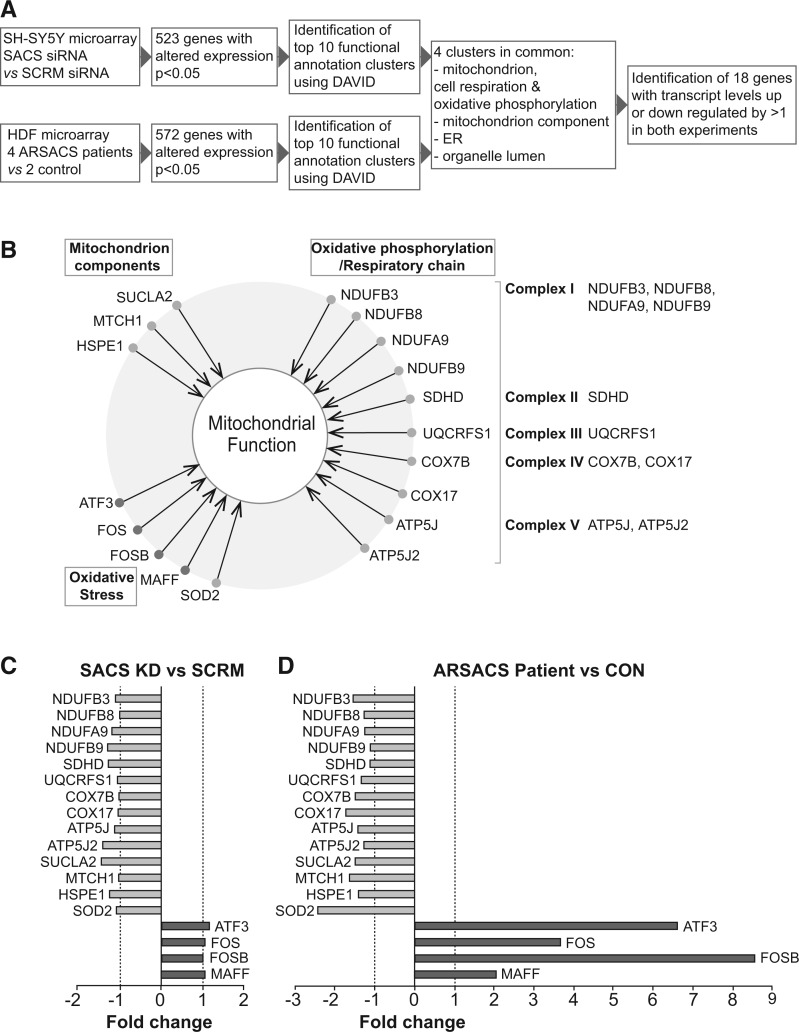
Microarray analyses identify altered expression of genes associated with mitochondrial function in cells with reduced levels of sacsin. (**A)** Schematic summary of microarray study design. Two microarray experiments were performed. The first compared gene expression in SH-SY5Y cells transfected with siRNAs targeting sacsin (*SACS*) and scrambled (*SCRM*) control siRNAs. The second experiment compared gene expression in ARSACS patient and WT control HDFs. Gene ontology analyses were used to identify functional clusters of genes that were altered in both experiments. (**B**) Functional clustering of mitochondrial genes identified as having altered expression by microarray analyses in both *SACS* knockdown SH-SY5Y and ARSACS patient HDFs. (**C**) Fold change in expression of mitochondrial genes identified through microarray analyses comparing SH-SY5Y cells transfected with siRNAs targeting sacsin and scrambled siRNAs. (**D**) Fold change in expression of mitochondrial genes identified through microarray analyses comparing ARSACS patient and WT control HDFs.

To elucidate pathogenic mechanisms of ARSACS, we performed two microarray experiments using a human Illumina Whole Genome Gene Expression BeadChip (HT-12 v4.0). We compared transcript levels in SH-SY5Y cells transfected with siRNAs targeting sacsin and control siRNAs (Supplementary Material, Figure S1), while in a complimentary experiment, gene expression profiling was performed to compare ARSACS patient and control human dermal fibroblasts (HDFs). For this part of the study, four ARSACS HDF lines and two control HDF lines were used. ARSACS HDFS were from three compound heterozygous patients (c.2094-2A > G/Q4054*; K1715*/R4331Q and R2002fs/Q4054*) and one homozygous patient (2801delQ). Immunoblotting of lysates from these ARSACS HDF lines was performed using a commercially available antibody against an undefined sequence between residues 4100 and 4200 of sacsin. This detected a sacsin band in cells homozygous for the single amino acid deletion mutation 2801delQ, which was of similar intensity to that seen in wild-type control HDFs. In cells with the K1715*/R4331Q mutation, a significantly fainter sacsin band was visible. This is consistent with the K1715*/R4331Q HDF line expressing detectable protein from a single allele. No sacsin was detected in the other two ARSACS HDF lines although we could not preclude expression of a truncated protein (Supplementary Material, Figure S2). In sacsin knockdown cells, 523 genes showed a significant change in expression levels, whereas in patient cells, expression of 572 genes was altered (*P* < 0.05) (Fig. 1A). Functional annotation clustering using the Database for Annotation, Visualization and Integrated Discovery (DAVID) (19), identified enrichment of genes in four clusters as common to the microarray comparing SH-SY5Y cells transfected with siRNAs targeting sacsin or scrambled siRNAs and the microarray comparing ARSACS and control HDFs (Fig. 1B). Two of these clusters related directly to mitochondria with gene ontology terms including mitochondrial components, cell respiration, oxidative phosphorylation, and oxidative stress. From the genes in these groupings, we identified 18 genes where transcript levels showed a 1-fold or greater upregulation or downregulation in both the sacsin knockdown cells (Fig. 1C) and ARSACS HDF cells (Fig. 1D). These included 10 genes that function in oxidative phosphorylation (*NDUFB3, NDUFB8, NDUFA9, NDUFB9, SDHD, UQCRFS1, COX7B, COX17, ATP5J* and *ATP5J2*), five genes linked to oxidative stress (*SOD2, MAFF, FOSB, FOS* and *ATF3*) and three genes listed as mitochondrial components (*SUCLA2, MTCH1* and *HSPE1*) (Fig. 1D).

### Loss of sacsin function causes alterations in levels of oxidative phosphorylation and oxidative stress gene transcripts

**Figure 2.**
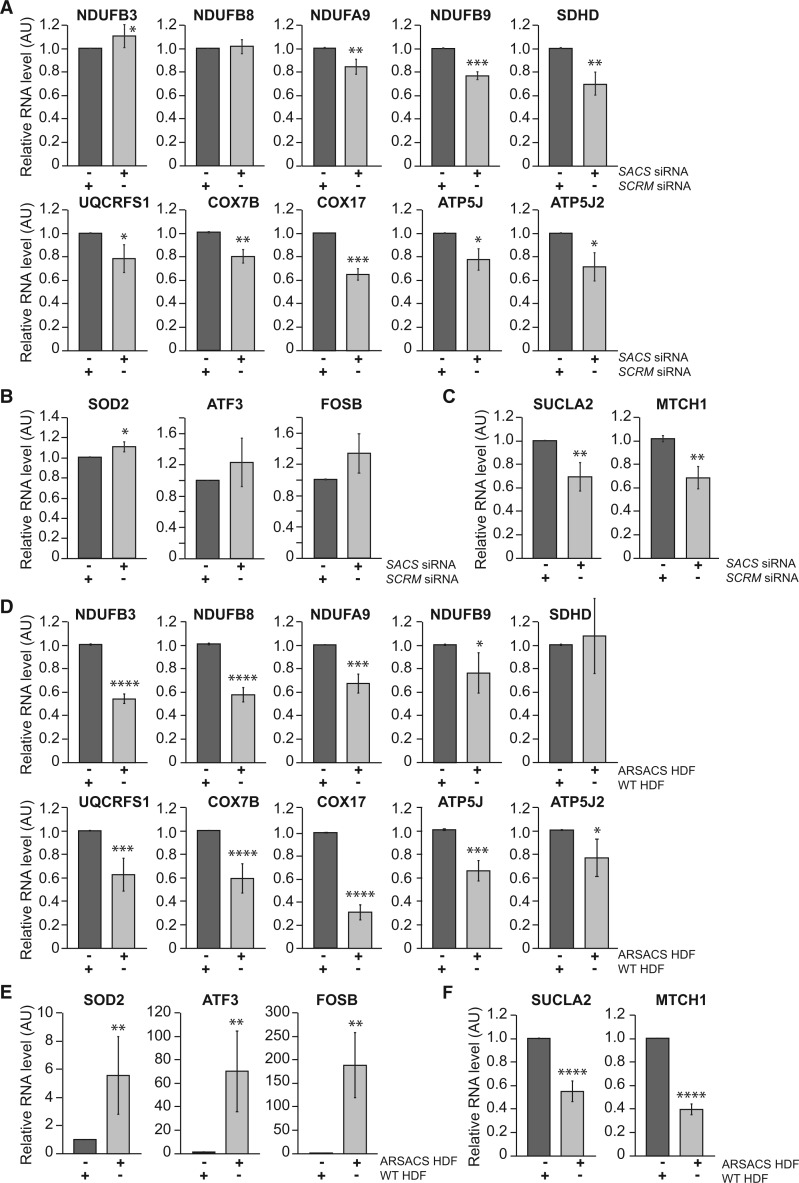
RT-qPCR analyses shows cells with reduced levels of sacsin have alterations in levels of oxidative phosphorylation and oxidative stress gene transcripts. Comparison of transcript levels of oxidative phosphorylation (**A**), oxidative stress (**B**), and mitochondrion component (**C**) genes between SH-SY5Y cells transfected with siRNAs targeting sacsin and scrambled siRNAs. RT-qPCR was performed on total RNA extracted from five separate knockdown *SACS* experiments. Transcript levels of the genes analyzed were normalized to GAPDH levels with data shown as mean fold change relative to cells transfected with scrambled siRNA (*SCRM*). Statistical significances determined by *t* test are indicated. Comparison of transcript levels of oxidative phosphorylation (**D**), oxidative stress (**E**), and mitochondrion component (**F**) genes between ARSACS patient and WT control HDFs. RT-qPCR was performed on total RNA extracted from four ARSACS HDF lines and five WT control HDF lines. Transcript levels of the genes analyzed were normalized to GAPDH levels with data shown as mean fold change relative to WT controls. Statistical significances were determined by Mann–Whitney *U* test. Error bars = SD. *P ≤ 0.05, **P ≤ 0.005, ***P ≤ 0.005.

To validate alterations in transcript levels of oxidative phosphorylation and oxidative stress genes, we used RT-qPCR. In an experiment that was independent from the microarray analyses, total RNA was extracted from sacsin knockdown and control SH-SY5Y cells. RT-qPCR was then performed for each gene of interest with raw data normalized to *GAPDH* levels. In agreement with the microarray data, we observed that expression of eight oxidative phosphorylation genes were significantly decreased in cells with reduced levels of sacsin (Fig. 2A). This included genes in each mitochondrial respiratory chain complex, with *COX17* showing the greatest reduction in transcript levels (>35%). Contrastingly, two oxidative stress genes *ATF3* and *FOSB* were slightly increased in sacsin knockdown cells although these changes were not significant (Fig. 2B). *SOD2*, which the microarray suggested was downregulated, was significantly upregulated in sacsin knockdown cells. Moreover, the mitochondrial component genes (*SUCLA2* and *MTCH1*) that were identified as having reduced transcript levels in the microarray experiment were confirmed as being downregulated by RT-qPCR (Fig. 2C).

Total RNA was also extracted from the four ARSACS patient HDF cell lines and five control HDF lines and RT-qPCR performed. Again we observed a downregulation of oxidative phosphorylation genes (Fig. 2D), with 9 of the 10 genes identified from the microarray analyses exhibiting significantly decreased transcript levels in ARSACS HDFs (the exception was *SDHD*). As observed in the knockdown experiment, *COX17* was the oxidative phosphorylation gene that showed the greatest reduction in transcript levels (>60%). ARSACS HDFs also exhibited an upregulation of oxidative stress genes (Fig. 2E) with *SOD2, ATF3* and *FOSB* transcripts all significantly increased relative to controls. The most dramatic change was for *FOSB*, which showed a more than 150-fold increase in transcript levels. The mitochondrial component genes were also confirmed as downregulated in the patient cells (Fig. 2F). With the exception of data for *SOD2*, changes in gene expression identified by the microarray and RT-qPCR experiments were in broad agreement. The combined data from analyses in sacsin knockdown cells and patient HDF demonstrate reduced expression of oxidative phosphorylation genes in cellular models of ARSACS and suggests that oxidative stress is increased.

### Bioenergetic parameters are altered in sacsin knockdown and ARSACS patient cells

**Figure 3.**
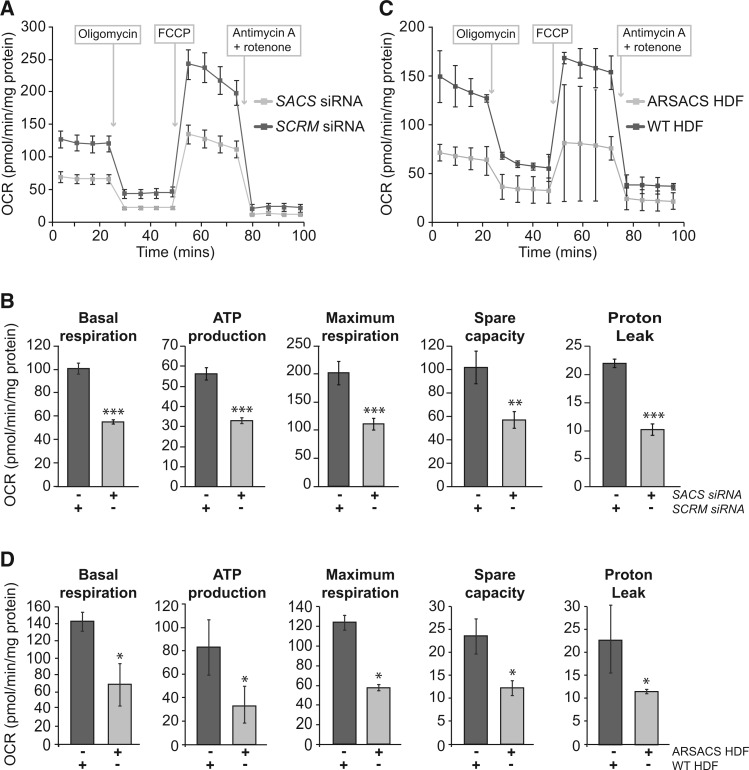
Bioenergetic function of mitochondria is impaired in sacsin knockdown and ARSACS patient cells. (**A**) Comparison of OCR between cells transfected with siRNAs targeting sacsin and control siRNAs. (**B**) Comparison of basal respiration, ATP production, proton leak, maximum respiration and spare capacity in cells transfected with siRNAs targeting sacsin and control siRNAs, were calculated from the mean of four time points. (**C**) Comparison of OCR between ARSACS patient and control cell lines. Measurements were made under basal conditions and after addition of oligomycin, FCCP and Antimycin A plus rotenone (arrows indicate the time points when drugs were added). (**D**) Comparison of basal respiration, ATP production, proton leak, maximum respiration and spare capacity in ARSACS patient and control HDFs, were calculated from the mean of four time points. For siRNA knockdown experiments n = 12 (12 independent transfection for *SACS* and *SCRM* siRNAs) and for ARSACS patient cells n = 4 (four patient and four control HDF lines). Statistical significances were determined by *t* test or Mann–Whitney *U* test as appropriate. Error bars = SD. *P ≤ 0.05, **P ≤ 0.005, ***P ≤ 0.005.

Bioenergetics profiling was performed to test for functional consequences of altered mitochondrial gene expression in cells lacking sacsin. Oxygen consumption rates (OCR) were measured in SH-SY5Y cells that had previously been transfected with siRNAs targeting sacsin or control siRNAs (Fig. 3A). Measurements were made under basal conditions and after sequential treatment with the ATP synthase (complex V) inhibitor oligomycin, followed by the uncoupling agent carbonyl cyanide-*4* (trifluoromethoxy) phenylhydrazone (FCCP). OCR was then determined after mitochondrial respiration was shut down by combined treatment with the complex I inhibitor rotenone and the complex III inhibitor antimycin A. These analyses revealed that basal respiration, ATP production, proton leak, maximal respiration, and spare capacity were all reduced in SH-SY5Y cells with reduced levels of sacsin, compared with controls (Fig. 3A and B). These key parameters of mitochondrial respiration were also compared in ARSACS patient HDF and control HDF lines. As observed in SH-SY5Y cells with reduced levels of sacsin, ARSACS patient HDF showed decreased OCR under basal conditions and after addition of mitochondrial inhibitors (Fig. 3C and D). Both knockdown cells and HDFs showed a significant reduction in ATP-coupled respiration and maximal respiration, strongly suggesting that compromised mitochondrial respiration is a feature of ARSACS.

### Increase levels of ROS in ARSACS patient cells

**Figure 4.**
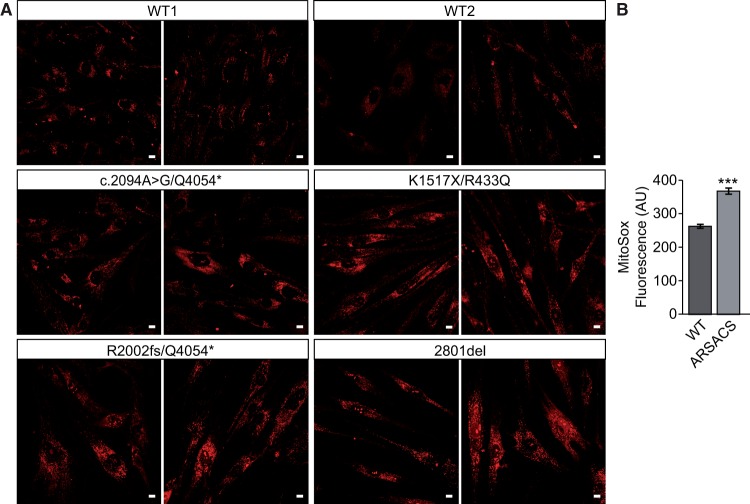
Levels of ROS are increased in ARSACS patient HDFs relative to controls. (**A**) Representative images of WT control and ARSACS patient HDFs stained the mitochondrial superoxide indicator MitoSOX (red). Live cell confocal imaging was used to generate maximum intensity projections. Scale bar = 10 µm. (**B**) MitoSOX fluorescent intensity was then quantified in individual cells from confocal maximum intensity projections (measurements were made in 25 cells in each patient and control line with three experimental replicates). Error bars = SEM. *P ≤ 0.05, **P ≤ 0.005, ***P ≤ 0.005.

As gene expression analyses suggested increased oxidative stress in cells with reduced levels of sacsin, we performed staining with MitoSOX Red to detect superoxide species. Confocal imaging of HDFs and subsequent measurement of fluorescent intensity revealed increased levels of MitoSOX staining in ARSACS patient cells relative to controls, this was confirmed by quantitative analyses of confocal images (Fig. 4). We were unable to validate these findings in sacsin knockdown SH-SY5Y cells, potentially because the transient knockdown did not reduce sacsin levels sufficiently, or for sufficient time, for ROS to accumulate (not shown).

### Localization of Drp1 foci to mitochondria is reduced in sacsin knockdown cells

The gene expression and functional data presented here support that mitochondrial health is impaired in ARSACS. Potential mechanisms for this could include an increase in the proportion of defective mitochondria in sacsin null cells as a consequence of impaired mitochondrial dynamics and quality control. Moreover, ARSACS-associated changes in mitochondrial network organization are consistent with impaired Drp1-mediated mitochondrial fission (3).

**Figure 5.**
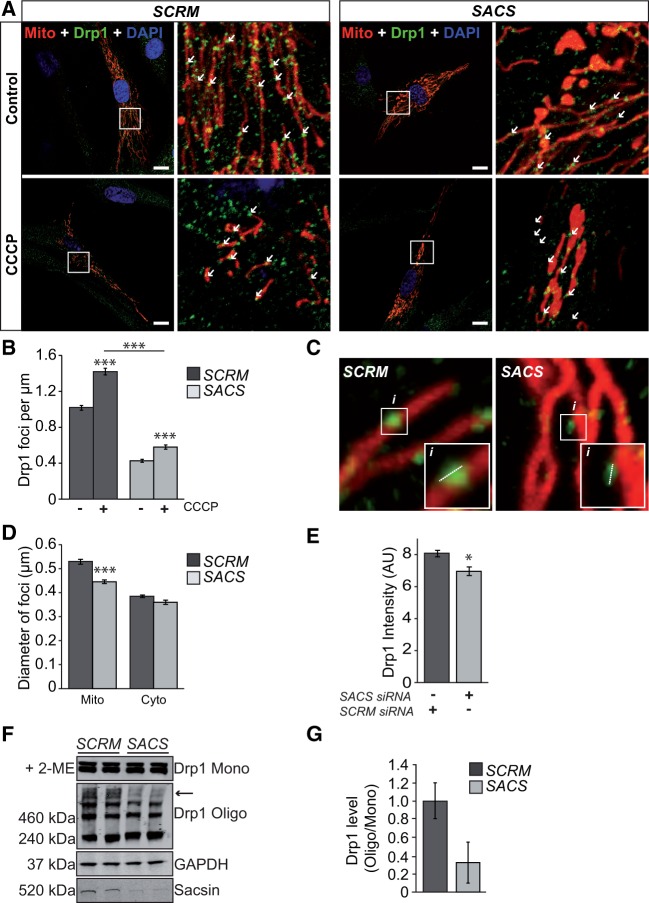
Localization of Drp1 to mitochondria is reduced in sacsin knockdown cells. (**A**) HDFs were cotransfected with control scrambled (*SCRM*) siRNA or siRNA targeting sacsin (*SACS*) and DSRed2-mito (red). After 48 hours cells were treated for 1 hour with 20μm CCCP and then processed for immunofluorescent detection of Drp1 (green) and counterstained with DAPI (blue) for nuclei. Examples of mitochondrial associated Drp1 foci are indicated by arrows. Scale bar = 10 µm. (**B**) The number of Drp1 foci that localized to mitochondria in *SCRM* and *SACS* cells with and without CCCP treatment were then quantified from confocal Z-stacks. This data was expressed as the number of Drp1 foci localized to mitochondria per 1 µm of measured length (quantification was performed from at least six mitochondria in 60 cells for each treatment, from three independent replicates). (**C**) Representative micrographs used to quantify the diameter (**D**) and fluorescent intensity (**E**) of mitochondrial associated and cytosolic localized Drp1 foci (at least 450 foci were measured from 18 cells, from three independent replicates). (**F**) Immunoblot showing high molecular weight Drp1 complexes are reduced in sacsin knockdown cells. SH-SY5Y cells were transfected with control scrambled (*SCRM*) siRNA or siRNA targeting sacsin (*SACS*). After 48 hours, cells were treated with the crosslinker DSP and lysed. Total cell lysates were blotted under reducing (+2-ME) and non-reducing conditions for Drp1. Levels of sacsin in SCRM and SACS cells were assessed by immunoblot for sacsin, whereas GAPDH was used as a loading control. Arrow indicates high molecular weight Drp1 species. (**G**) Densitometry analyses of immunoblots were used to quantify the ratio of high molecular weight Drp1 complexes relative to monomeric Drp1 (n = 5). Statistical significances were determined by *t* test. Error bars = SEM. *P ≤ 0.05, **P ≤ 0.005, ***P ≤ 0.005.

Based on these data, we investigated mitochondrial recruitment of Drp1 in sacsin knockdown cells. Here, we initially used control HDFs as they have a more distributed mitochondrial network that was better for quantitative imaging of Drp1 association. Cells were transfected with DSRed2-mito along with scrambled siRNAs or siRNAs targeting *SACS*. After 48 hours, cells were fixed and stained for Drp1. First, we confirmed that sacsin knockdown in HDFs impacted mitochondrial network organization, observing that relative to control cells, sacsin knockdown HDFs frequently exhibited a hyperfused mitochondrial phenotype indicated by balloon-like or bulbed mitochondria (Supplementary Material, Figure S3). Interestingly, we also observed a larger total mitochondrial volume in individual cells transfected with *SACS* siRNAs relative to controls, suggesting increased mitochondrial mass in response to loss of sacsin (Supplementary Material, Figure S3). We then analyzed the number of Drp1 foci localized on individual mitochondria (Fig. 5A and B). We observed that for every 1 µm along the length of a mitochondrion, an average of 0.81 ± 0.047 Drp1 foci occurred. This incidence of mitochondrial-associated Drp1 foci was significantly reduced in sacsin knockdown HDFs, where only 0.68 ± 0.039 foci localized per 1 µm of mitochondrion.

We next tested whether this observation reflected a reduction in the capacity of mitochondria in sacsin-deficient cells to recruit Drp1 to prospective sites of fission (Fig. 5A and B). To induce fission, siRNA-transfected cells were treated with carbonyl cyanide *m*-chlorophenyl hydrazine (CCCP), which abolishes membrane potential across the inner mitochondrial membrane leading to Drp1-dependent fragmentation of mitochondria and ultimately mitophagy (20). Confocal imaging indicated extensive mitochondrial fragmentation after CCCP treatment in the cells transfected with the scrambled siRNA relative to cells where *SACS* was targeted (Fig. 5A). The degree of mitochondria fragmentation was analyzed by comparing the number of individual mitochondrion in cells treated with CCCP and vehicle controls. This confirmed that CCCP treatment resulted in higher levels of fragmentation of mitochondrial network in control HDFs than sacsin knockdown cells (Supplementary Material, Figure S3). Quantitative analyses identified that treatment with CCCP resulted in a significant increase in mitochondrial localized Drp1 foci in scrambled siRNA controls (*P* < 0.0001). The number of Drp1 foci at mitochondria also increased in sacsin knockdown cells, but to a lesser degree than for the control (Fig. 5B). Moreover, sacsin knockdown cells treated with CCCP still had less mitochondrial localized Drp1 foci than control cells that had not been treated to induce fission. This reduction in Drp1 recruitment was also confirmed in siRNA-treated SH-SY5Y cells (Supplementary Material, Figure S4).

As reduced cellular levels of sacsin impaired Drp1 recruitment to mitochondria, we hypothesized that the amount of Drp1 in individual foci may also be affected. To investigate this further, we measured the diameter of individual cytosolic and mitochondrially localized Drp1 foci in control and sacsin knockdown cells (Fig. 5C and D). In control cells, we observed that cytosolic Drp1 foci were significantly smaller than those localized to mitochondria (*P* < 0.0001), with respective diameters of 0.38 ± 0.0053 µm and 0.52 ± 0.0094 µm. Comparison of the diameter of Drp1 foci between cells transfected with scrambled and *SACS* siRNA revealed that mitochondrial associated foci were 16% smaller in cells lacking sacsin (*P* < 0.0001). In contrast, there was no significant change in the size of cytosolic Drp1 foci between control and sacsin knockdown cells. We also measured the intensity of the Drp1 fluorescent signal for the mitochondrial associated Drp1 foci, observing that this was also significantly reduced in sacsin knockdown cells relative to controls (*P* < 0.01) (Fig. 5E). To further confirm that Drp1 localization to mitochondria was reduced upon sacsin knockdown, we examined levels of the fission factor in mitochondrial fractions generated from SH-SY5Y cells. Immunoblot analyses of total cell lysates and mitochondrial fractions further supported reduced levels of mitochondrial-associated Drp1 in cells with reduced levels of sacsin (Supplementary Material, Figure S4).

A potential explanation for the reduction in mitochondrial-associated Drp1 was that total levels of the fission factor are reduced in sacsin knockdown cells. Immunoblot analyses in SH-SY5Y suggested that this was not the case, as sacsin knockdown did not significantly alter total Drp1 levels (Fig. 5F and Supplementary Material, Figure S4). However, when cells transfected with scrambled siRNAs and siRNAs targeting *SACS* were treated with a cross-linking agent before lysates were immunoblotted for Drp1, we observed a reduction in higher molecular weight Drp1 species in sacsin knockdown cells (Fig. 5F and G). This may reflect an absence of Drp1:sacsin complexes, but could also indicate that Drp1 higher order oligomers or complexes between Drp1 and other components of the fission machinery are reduced.

Interestingly, as Drp1-mediated fission may precede removal of damaged mitochondria by mitophagy, we saw some evidence for decreased mitochondrial turnover in sacsin knockdown SH-SY5Y cells. Using the mitochondrial matrix targeted fluorescent protein MitoTimer (21), we cotransfected a Tet-On SH-SY5Y cell line with pTRE-tight-MitoTimer and control siRNAs or siRNAs targeting *SACS*. Twenty-four hours after transfection, cells were treated with doxycycline for 1 hour to induce the expression of the MitoTimer protein. A further 24 and 48 hours after this treatment, cells were imaged and levels of fluorescence were quantified. This showed a significant greater decrease in MitoTimer red fluorescence in control cells relative to sacsin knockdown cells (Supplementary Material, Figure S5).

### Localization of Drp1 foci to mitochondria is reduced in ARSACS patient fibroblasts

**Figure 6.**
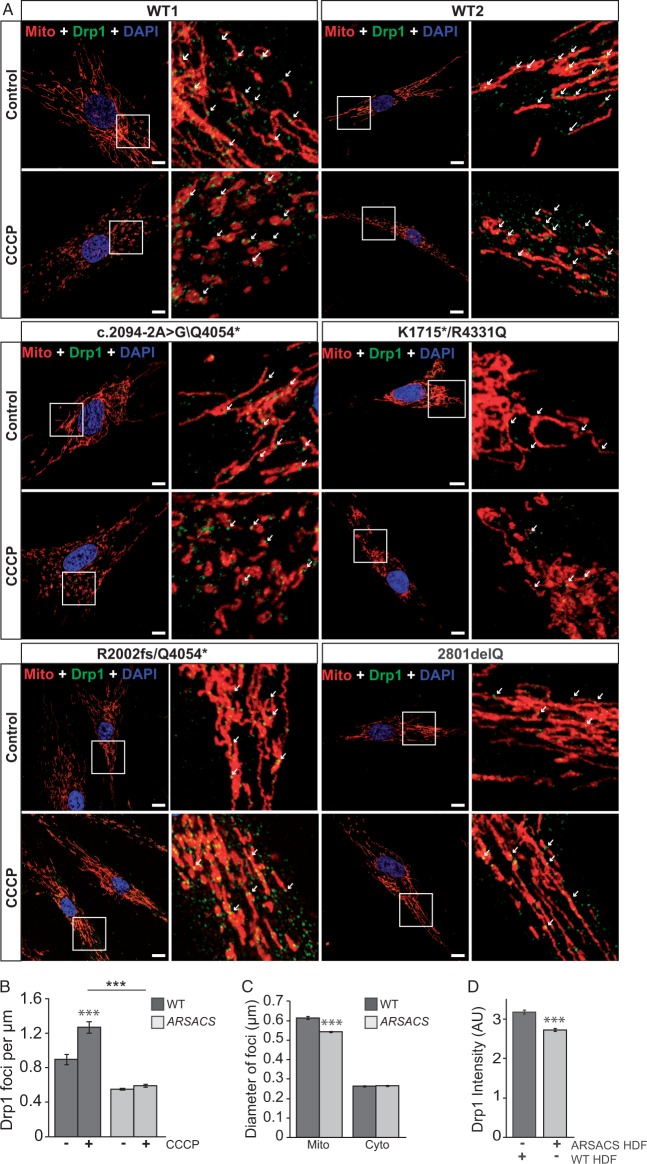
Localization of Drp1 to mitochondria is reduced in ARSACS patient cells. (**A)** Control and patient HDFs were treated for 1 hour with CCCP and then processed for immunofluorescent detection of Tom20 (red) and Drp1 (green). Cells were also counterstained with DAPI (blue) to detect nuclei. Representative images are shown for two control lines (WT1 and WT2) and each ARSACS patient line used in this study. Examples of mitochondrial-associated Drp1 foci are indicated by arrows. Scale bar = 10 µm. (**B**) The number of Drp1 foci that localized to mitochondria in control and patient HDFs with and without CCCP treatment were then quantified from confocal Z-stacks. These data were expressed as the number of Drp1 foci localized to mitochondria per 1 µm of measured length (using four patient and four control lines quantification was performed from at least six mitochondria in 45 cells for each treatment, from three independent replicates). (**C** and **D**) Quantification of the diameter (C) and fluorescent intensity (D) of mitochondrial associated and cytosolic localized Drp1 foci (at least 500 foci were measured from 18 cells, from three independent replicates). Statistical significances were determined by Mann–Whitney *U* test. Error bars = SEM. *P ≤ 0.05, **P ≤ 0.005, ***P ≤ 0.005.

As a precursor to investigating Drp1 recruitment in ARSACS HDFs, we established that altered mitochondrial network morphology was a common feature of all ARSACS patient HDF lines investigated (Supplementary Material, Figure S6). This was accompanied by an increase in total mitochondrial volume per cell that was significant in two of the ARSACS HDF lines, but not overall (Supplementary Material, Figure S6). As in sacsin knockdown cells, we quantified the number of Drp1 foci localized on individual mitochondria in patient and control lines. This included a comparison of the number of Drp1-associated mitochondrial foci between vehicle-treated cells and cells treated with CCCP to induce unopposed Drp1 recruitment (Fig. 6A). We observed that for all the patient cell lines examined, significantly less Drp1 foci were localized to mitochondria than for control cells (Fig. 6B and Supplementary Material, Figure S7). ARSACS patient cells also showed a reduced capacity to increase the number of mitochondrial-associated foci after the induction of fission with CCCP. Mitochondrial-associated Drp1 foci were also smaller with a reduced fluorescent intensity (when detected by immunofluorescent staining for Drp1), relative to controls (Fig. 6C and D).

In combination, our data in sacsin knockdown cells and ARSACS HDF lines, indicate that loss of sacsin impairs mitochondrial association of Drp1.

## Discussion

Data presented here support that decreased mitochondrial function is a feature of ARSACS. Specifically, we show that oxidative phosphorylation is impaired in cells lacking sacsin, including ARSACS patient HDFs. This is also consistent with decreased expression of nuclear genes encoding respiratory chain complex components and other mitochondrial proteins. These observations combined with an increase in expression of oxidative stress gene transcripts demonstrate that mitochondrial health is reduced in ARSACS. This is in agreement with another very recent study that has also reported reduced bioenergetic function and oxidative stress in ARSACS (22). This study compared five patient and control HDFs and did not corroborate findings in any cellular models with isogenic controls (this was a key reason we also performed comparative analyses in sacsin knockdown cells).

Mitochondrial dynamics are disrupted in ARSACS, with our data showing that patient HDFs with a range of different ARSACS mutations have disrupted mitochondrial network organization. This is consistent with the finding that recruitment of the fission protein Drp1 to the OMM is reduced in both *SACS* knockdown cells and ARSACS patient HDFs. As fission is integral to mitochondrial quality control, we hypothesize that a failure to remove damaged mitochondria could explain the reduction in mitochondrial respiration and thus contribute to the molecular pathology of ARSACS. This idea is supported by our observation that mitochondrial turnover, measured by loss of MitoTimer fluorescence after a pulse of expression, is reduced in sacsin knockdown SH-SY5Y cells. In the context of this hypothesis, it is important to consider known consequences of impaired Drp1 function. These include that complete loss of Drp1 is embryonic lethal in mice, with fibroblasts from these animals having elongated and more interconnected mitochondria (23,24). In humans Drp1 also appears to be an essential protein as a mutation at a conserved residue (A359D) in the middle domain of Drp1 was neonatal lethal. In this case, fibroblasts again exhibited a phenotype with mitochondrial elongation with tubular structures concentrated around the nucleus (25). Loss of sacsin is not embryonic lethal in mice or humans and Drp1 association with mitochondria is reduced rather than abolished in sacsin null cells. This less severe ARSACS phenotype signifies that sacsin is not essential for Drp1-mediated fission, but may be required for normal regulation of mitochondrial division in some cell types.

This might be more similar to the phenotype in cells depleted for Fis1 and Mff (26). Targeting of these integral OMM proteins that act as receptors to recruit Drp1 to the mitochondrial surface were shown to result in increased mitochondrial interconnectivity with a reduction in the number and size of mitochondrial associated Drp1 foci. Despite phenotypic overlap, it seems unlikely that sacsin would function like Fis1 and Mff as a component of the Drp1 receptor machinery. This is because sacsin is a cytosolic protein that localizes at or near the OMM through an unknown mechanism.

Another possible explanation for the decreased association of Drp1 to mitochondria in cells with less sacsin is impaired traffic of Drp1. Modulators of Drp1 distribution include the cytoplasmic dynein machinery, with overexpression of the dynactin subunit, dynamitin (p50), altering mitochondrial dynamics and reducing the association of Drp1 with mitochondria (27).

It also seems likely that cytoskeletal disorganization more broadly contributes to the ARSACS mitochondrial phenotype. Loss of sacsin results in the accumulation of non-phosphorylated neurofilaments in vulnerable neuronal populations in *SACS*^-/-^ mouse and ARSACS patient brain (4). Given that intermediate filaments, actin microfilaments and microtubules form an interlinked cytoskeletal network, it is likely that disruption of neurofilament organization would impact directly on the distribution of mitochondria and plausibly influence Drp1 dynamics. Interestingly, connections between Drp1 mitochondrial association and cytoskeletal abnormalities have been established in animal models of Alzheimer’s disease and related tauopathies. Specifically, mitochondrial elongation as a consequence of Drp1 mislocalization has been reported in *Drosophila* and mouse neurons expressing human tau, with evidence that this is by a mechanism where phosphorylated tau excessively stabilizes actin, inhibiting F-actin and myosin-dependent translocation of Drp1 and mitochondria, with this contributing to mitochondrial dysfunction and cell death (28).

It is interesting that patient HDFs have detectable cellular phenotypes, yet ARSACS is purely a neurodegenerative disease. This supports the hypothesis that abnormal mitochondrial dynamics and function is particularly detrimental to neurons. Explanations of why neurons are highly sensitive to perturbations in mitochondrial dynamics include that they have energy requirements away from the cell body and must be able to traffic mitochondria relatively long distances (12). For ARSACS, impaired traffic is supported by the observation that mitochondrial motility was significantly reduced in axons of *SACS*^−/− ^mouse motor neurons (4). Clues to potential mechanisms of Purkinje cell death, in response to impaired fission, come from studies using mouse postmitotic Purkinje cells depleted for Drp1. In these cells, elongated mitochondria ultimately become swollen due to oxidative damage, losing respiratory function and accumulating markers of degradation. Moreover, treatment with antioxidants was shown to be neuroprotective (29). In addition to neurons being particularly vulnerable to aberrant mitochondrial dynamics, they are reported to have very limited glycolysis (30). Relative to some other cell types, this makes neurons more highly dependent on aerobic oxidative phosphorylation, which our microarray and bioenergetics data indicate is impaired when sacsin function is lost.

Ultimately, it will be important to analyze the degree of mitochondrial dysfunction associated with ARSACS in neurons. It is encouraging that ARSACS HDFs have robust and quantifiable mitochondrial phenotypes, as this suggests that induced pluripotent stem cell iPSC-derived neurons generated from fibroblasts will be a useful and more disease relevant model.

It has previously been found that oxidative phosphorylation complex assembly and function is modulated by mitochondrial dynamics, including Drp1 mediated fission (31). Moreover, gene expression studies have shown that levels of gene transcripts associated with oxidative phosphorylation, and other aspects of mitochondrial bioenergetics, are often reduced in neurodegenerative disease (32,33). The expression of nuclear genes involved in mitochondrial function, including those encoding proteins required for oxidative phosphorylation, is controlled by mitochondria-to-nucleus signalling mechanisms. These in turn are modulated by factors such as calcium, ATP and ROS levels (34). It is thus likely that the changes in gene expression observed in response to loss of sacsin are a consequence of the altered mitochondrial dynamics either directly or more indirectly through factors such as the accumulation of ROS.

Although we have shown reduced localization of Drp1 to the OMM contributes to altered mitochondrial dynamics in ARSACS, the function of sacsin remains unclear. Based on sacsin’s repeating structure and large size, one possibility is that it may have a scaffold function. As sacsin has multiple domains that link to molecular chaperone and protein degradation systems, it could potentially tether molecules together in a chaperone-regulated system. Alternately, sacsin may function as a chaperone for a specific client or group of client proteins with the possibility that client protein misfolding affects Drp1-mediated fission through loss or gain of function. This would be partly analogous to common neurodegenerative diseases where both aberrant protein folding and disrupted mitochondrial dynamics are features.

## Materials and Methods

### Cell culture and sacsin knockdown

SH-SY5Y cells were from the American Type Culture Collection and were grown in Dulbecco’s Minimum Eagle Medium (DMEM) at a 1:1 ratio with Ham’s F12 medium. Cells were maintained in medium supplemented with 10% heat-inactivated foetal bovine serum (FCS) containing 100 U ml^−^^1^ penicillin and 100 mg ml^−1^ streptomycin. ARSACS patient fibroblasts were a gift from Dr Sascha Vermeer and colleagues at Radboud University Nijmegen Medical Centre (Nijmegen, Netherlands). These cells were collected as a part of a project approved by the Medical Ethics Committee of the Radboud University (CMO-nr 2014/155). Written informed consent to participate in this study was obtained from all patients. Control HDFs were purchased from PromoCell (Heidelberg, Germany) or were kindly provided by Dr Tristan McKay (Biomedical Sciences, St George’s, University of London), or Dr Sascha Vermeer. Control and ARSACS HDF lines used in this work were not closely age or sex matched, but were all between passage 3 and 8. HDFs were cultured in DMEM supplemented with 10% FBS and 50 U ml^−1^ penicillin and 50 μg ml^−^^1^ streptomycin (final concentration in media 1%). All cells were kept in a constant humidified atmosphere of 5% CO_2_ at 37°C. Cell culture reagents were from Life Technologies (Paisley, UK). To induce mitochondrial fission cells were treated for 1 hour with 20 µm CCCP, in their standard culture medium.

For sacsin knockdown a combination of three previously validated siRNAs targeting exons 6 (sense: GGAUGAUCCUC UGAAGGUC), 7 (sense: GCGGCCGAAUUCUAUAAAG) and 9 (sense: CGUAAGAUUUCUAGAUGAC) of *SACS* were used (3,6). These siRNAs were at a concentration of 10 nm each and were transfected in combination using Lipofectamine 3000 (ThermoFisher Scientific, Paisley, Scotland), according to the manufacturers instructions. A negative control siRNA that has no significant sequence similarity to human gene sequences was used as a control at a concentration of 30 nm.

### Microarrays and RT-qPCR

Total RNA was isolated from SH-SY5Y cells (48 hours post transfection with siRNAs) or from HDFs using the RNeasy Mini kit (Qiagen, Hilden, Germany). RNA samples were then reversed transcribed using the QuantiTect Reverse Transcription Kit (Qiagen) to give cDNA. Gene expression profiling using cDNA from SH-SY5Y or HDFs was then performed using a HumanHT-12 v4 Expression BeadChip arrays (Illumina, San Diego, USA). All arrays were hybridized at 58 °C for 16–20 h, followed by wash and stain procedures according to the Direct Hybridization Assay Guide (Illumina). Fluorescent signals were obtained by scanning with iScan System, and data were extracted with Gene Expression Module 1.0.6 in GenomeStudio Software 2011.1 (Illumina) with or without background subtraction. Pathway and ontology analysis were performed using DAVID (The Database for Annotation, Visualization and Integrated Discovery) v6.7, which enabled functional annotation clustering (19). RT-qPCR was performed using the MX3000p QPCR Systems (Agilent, Santa Clara, USA). PCR reactions contained 100 ng of template cDNA, 200 nm of each gene specific primer, 5 µl of qPCRBIO SyGreen Mix (PCR Biosystems, London, UK) and were made to a total volume of 10 µl with ultra-pure water. Relative gene expression was calculated using the 2(-Delta Delta C(T)) (ΔΔCT) method where GAPDH was used as the reference gene (35). That GAPDH transcript levels in sacsin knockdown SH-SY5Y cells were not significantly different from controls was confirmed by RT-qPCR using 18S RNA levels for normalization (not shown). PCR conditions and primer sequence for *NDUFB3, NDUFB8, NDUFA9, NDUFB9, SDHD, UQCRFS1, COX7B, COX17, ATP5J, ATP5J2, SOD2, MAFF, FOSB, FOS, ATF3, SUCLA2, MTCH1, HSPE1* and *GAPDH* are available upon request.

### Oxygen consumption rate measurement

Oxygen consumption rate was measured using an XF Extracellular Flux Analyser (Seahorse Bioscience, MA, USA). SH-SY5Y and HDFs were seeded at densities of 50 000 and 300 000 cells per well, respectively. Cells were cultured on Seahorse XF-96 microplates and allowed to grow overnight. On the day of metabolic flux analysis, cells were changed to unbuffered DMEM (DMEM base medium supplemented with 10 mm glucose, 1 mm sodium pyruvate, 2 mm L-glutamine, pH 7.4) and incubated at 37 °C in a non-CO_2_ incubator for 1 hour. All medium and injection reagents were adjusted to pH 7.4 on the day of assay. Baseline measurements of OCR (measured by oxygen concentration change) and extracellular acidification rate (measured by pH change) were taken before sequential injection of treatments/inhibitors: oligomycin (ATP synthase inhibitor, 4 µm), FCCP (mitochondrial respiration uncoupler, 1 µm), and antimycin A (Complex III inhibitor, 1 µm) in conjunction with rotenone (Complex I inhibitor, 1 µm). Four measurements over time were collected for each condition with 10–12 replicates per sample. Data were normalized to protein concentration (measured by Bradford assay).

### Detection of ROS

For detection of ROS, cells were seeded in 35 mm dishes with a glass insert designed for live cell imaging (MatTek Corporation, Ashland, USA), They were then stained with MitoSOX Red when 80% confluent. For staining, MitoSOX Red was diluted to a final concentration of 5 μm solution in phosphate buffer saline (PBS) containing 100 mm calcium and magnesium (PBS/Ca/Mg). Cells were washed once in PBS/Ca/Mg prior to MitoSOX addition and three further times after a 10-minute incubation (at 37°C in a 5% CO_2_ environment) with the dye. For imaging, dishes were placed on a 37 °C heated stage, and Z-stacks were collected using the 40× objective of a Zeiss LSM510 laser scanning confocal microscope (Zeiss, Jena, Germany). Fluorescent intensity was measured using the LSM510 Zen software, which allowed for the measurement of fluorescent intensity within a selected region of interest. All confocal image acquisition settings were constant throughout the experiment, including for comparison between patient and control cells.

### Immunofluorescent detection and staining

Immunofluorescent staining was as described previously (3). Briefly, cells cultured on glass coverslips were fixed with 4% formaldehyde for 15 minutes and then permeablized for 5 minutes with 0.2% Triton-X 100. Cells were incubated with primary antibodies for 2 hours in 0.02% Triton-X100, 1% bovine serum albumin and 10% normal goat serum, prior to washing and incubation with fluorescently labelled secondary antibodies (Alexa Fluor 488-conjugated goat anti-rabbit or Alexa Fluor 543 conjugated goat anti-mouse; ThermoFisher Scientific). Cells were then counterstained with DAPI and coverslips mounted for microscopy. Primary antibodies were used at the following titres: 1:500 for rabbit polyclonal anti-Tom20 (Santa Cruz Biotechnology) and 1:100 for monoclonal anti-Drp1 (BD Transductions, Oxford, UK). For staining of mitochondria with MitoTracker (ThermoFisher Scientific), the stock solution was diluted to a concentration of 100 nm in cell culture media prior to addition to cells for 30 minutes at 37°C in 5% CO_2_ atmosphere. After the incubation period, cells were washed twice cell culture media prior to live imaging or fixation. Confocal microscopy was performed using a LSM510 with a 63× objective.

### Morphometric analyses of mitochondrial networks

Morphometric analyses of mitochondrial network organization was performed on live cells as described previously (3). For volumetric analyses, confocal Z-stacks of MitoTracker stained cells were collected and used to generate maximum intensity projections. Surface rendered 3D images generated using the Surpass module of the Imaris image analysis software (Imaris 7.6.1 Bitplane, Concord, USA) were then used to calculate mitochondrial number and volume. Image acquisition settings and thresholding was consistent across datasets.

### Quantitative analyses of Drp1 localization

The number of Drp1 foci per micrometre of mitochondrial length was determined using the line trace function of the LSM510 Zen confocal software. Line trace measured the intensity of channel used for detection of mitochondrial and Drp1 fluorescent staining along individual mitochondria. The number of Drp1 foci along the measured length of mitochondria was then collated from the graphical and tabular output. Drp1 foci intensity and diameter were measured from confocal images using a combination of the Surpass module and MeasurementPro modules of Imaris image analysis software. The mean intensity and diameter of Drp1 were quantified on randomly chosen mitochondria and in adjacent cytosolic regions in randomly chosen cells from three separate experiments. Area selection was blind to experimental status.

### Detection of Drp1 complexes

To study the effect of sacsin knockdown on Drp1 complex formation, SH-SY5Y cells were transfected with either SCRM or SACS siRNA, and 48 hours post-transfection cells were subjected to chemical cross-linking. The cleavable, homo-bifunctional cross-linker dithiobis[succinimidylpropionate] (DSP; Pierce) was added to the cultured cells and incubated for 1 hour at room temperature. Cross-linking was stopped by addition of Tris (pH 7.5) to a final concentration of 20 mm. Cells were then lysed and an equal volume of 2× SDS-PAGE sample buffer, without the reducing agent 2-Mercaptoethanol (2-ME), was added. Drp1 was detected with monoclonal anti-Drp1 at a titre of 1:500. GAPDH was also immunoblotted as a loading control with a rabbit anti-GAPDH at a titre of 1:5000 (Abcam).

### Mitochondrial turnover

Tet-On SH-SY5Y cells (a gift from Michael Cheetham, University College London), grown on glass coverslips, were co-transfected with pTRE-tight-MitoTimer (21) and control siRNAs or siRNAs targeting *SACS*. Twenty-four hours after transfection, cells were transferred to media containing 2 μg ml^−^^1^ doxycycline for 1 hour. After 1 hour, doxycycline was removed and cells were washed before being returned to normal media. Cells were subsequently fixed and prepared for confocal imaging. This was done 24 and 48 hours after the doxycycline pulse. Confocal Z-stacks were collected blind to experimental status and levels of mitochondrial MitoTimer fluorescence analyzed from maximum intensity projections. Image acquisition settings and thresholding was consistent across datasets. MitoTimer fluorescent protein has a time-dependent transition from green to red fluorescent emission over time [this has previously been shown to take 48 hours in HEK-293 cells (21)]. Consistent with this, and that we achieved a short period of MitoTimer expression, green fluorescence was not at quantifiable levels at 48 hours post the doxacycline treatment in control or sacsin knockdown cells.

### Statistical analyses

Statistical significance was determined by a Student’s *t*-test in the sacsin knockdown dataset and ANOVA or Mann–Whitney *U* test in the ARSACS patient HDF dataset. 

## Supplementary Material

Supplementary Material is available at *HMG* online.

*Conflict of Interest statement.* None declared.

## Funding

Biotechnology and Biological Sciences Research Council (BBSRC) [BB/L02294X/1], Fondation de l’Ataxie Charlevoix-Saguenay and Barts and the London Charity [417/1699]. Funding to pay the Open Access publication charges for this article was provided by Research Councils UK (RCUK). 

## Supplementary Material

Supplementary Data
