# Supplementary material for: A reduction in Drp1-mediated fission compromises mitochondrial health in autosomal recessive spastic ataxia of Charlevoix Saguenay
Source: Hum Mol Genet. 2016 Jun 10;25(15):3232–44. doi: 10.1093/hmg/ddw173 (PMC5179924; doi:10.1093/hmg/ddw173)
Supplement: Supplementary Data [file supp_25_15_3232__index.html]

A reduction in Drp1-mediated fission compromises mitochondrial health in autosomal recessive spastic ataxia of Charlevoix Saguenay — A reduction in Drp1-mediated fission compromises mitochondrial health in autosomal recessive spastic ataxia of Charlevoix Saguenay — Supplementary Data 

# A reduction in Drp1-mediated fission compromises mitochondrial health in autosomal recessive spastic ataxia of Charlevoix Saguenay

## Supplementary Data

files

- Supplementary Data - pdf file
