# Supplementary material for: A reduction in Drp1-mediated fission compromises mitochondrial health in autosomal recessive spastic ataxia of Charlevoix Saguenay
Source: Hum Mol Genet. 2016 Jun 10;25(15):3232–44. doi: 10.1093/hmg/ddw173 (PMC5179924; doi:10.1093/hmg/ddw173)
Supplement: Supplementary Data [file supp_ddw173_Bradshaw_et_al_supplemental_revised.pdf]

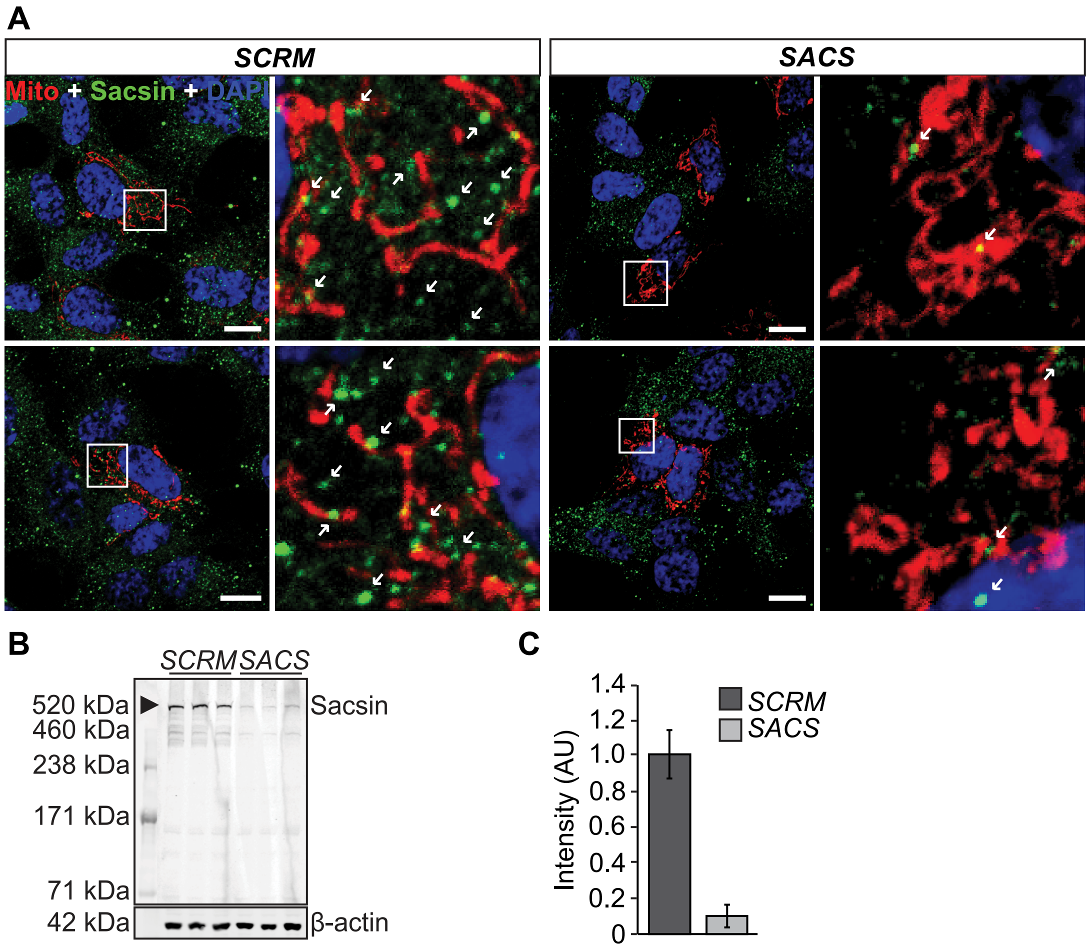

Figure S1. Confirmation of efficient siRNA-mediated knockdown of sacsin. (A) SH-SY5Ys were cotransfected with control scrambled (*SCRM*) siRNA or siRNA targeting sacsin (*SACS*) and DSRed2-mito (red). After 48 hours cells were stained for sacsin (green) and confocal imaging performed. The siRNAs were in excess to the DSRed2-mito plasmid in the transfection (molar ratio 168,000:1), such that we assumed any cell expressing DSRed2-mito would also contain the siRNAs. Confocal imaging demonstrated sacsin staining (indicated by arrows) was reduced in transfected cells. **(B)** Cell lysates were also generated from scrambled siRNA and sacsin siRNA transfected cells with levels of knockdown assessed by immunoblotting. β-actin used as a loading control. **(C)** Quantitative analyses by densitometry indicated that 48 hours post transfection cellular levels of sacsin were reduced by more than 80%. The anti-sacsin used was a commercial antibody that binds an unknown peptide between residues 4100 and 4200 of human sacsin.

A

ARSACS patient	Age at onset (yrs)	Disease duration (yrs)	Disease stage	SARA total score (0-40)	*SACS* mutations	Type of mutation
1	1	39	3	23.5	Q4054* & c.2094-2A>G	Nonsense/Splice-site
2	3	33	3	23	R2002fs & Q4054*	Frameshift/Nonsense
3	1	25	3	19.5	p.2801delQ	In frame deletion
4	8	45	3	21	K1715* & R4331Q	Nonsense/Missense

B

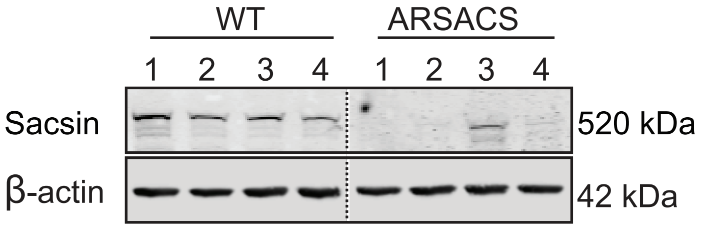

Figure S2. Phenotype of ARSACS HDF cell lines used in this study. (A) Summary of phenotypic characteristics of ARSACS patients who provided HDFs for this study. All patients were clinically examined by a neurologist using the Scale for the Assessment and Rating of Ataxia (SARA). This is an eight item scale that provides a comprehensive rating of simple ataxia tests including the following items: gait, stance, sitting, speech disturbance, finger chase, nose-finger test, fast alternating movements and heel-shin slide. All patients showed severe gait spasticity. All patients displayed severe lower limb spasticity except for patient 3 who showed moderate lower limb spasticity but with a mild upper limb spasticity that was not seen in the other 3 patients. They all displayed a sensomotor axonal neuropathy on EMG, with a secondary demyelination occurring in patient 4. **(B)** Immunoblot analyses of lysates from four WT control and four ARSACS HDF cell lines. Patient numbers correspond with those in the table (A). Lysates were also probed with β-actin as a loading control. Error bars = SD

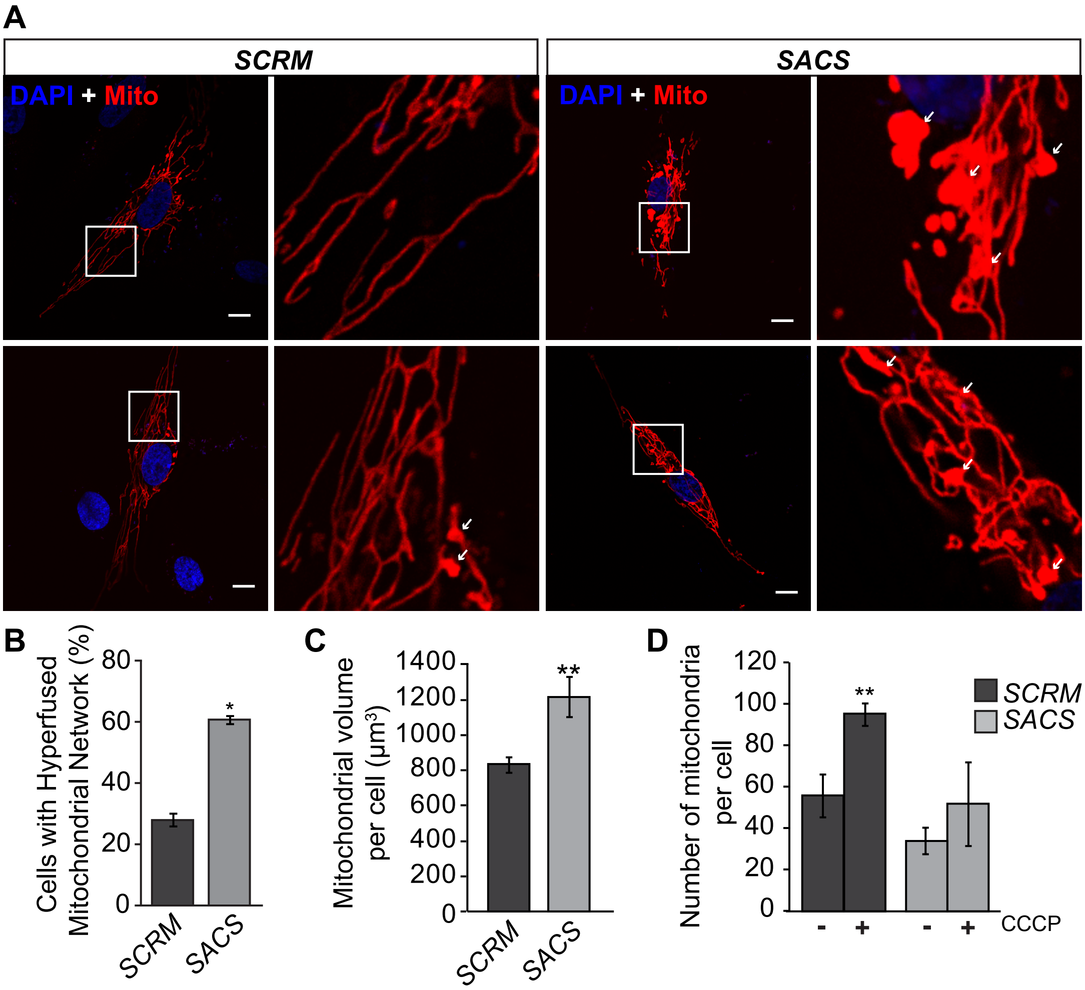

Figure S3. Sacsin knockdown HDFs have a hyperfused phenotype. (A) HDFs were cotransfected with control scrambled (*SCRM*) siRNA or siRNA targeting sacsin (*SACS*) and DSRed2-mito (red). Confocal imaging was then performed, with representative images shown. Scale bar = 10µm. **(B)** Incidence of cells containing hyperfused mitochondria was then quantified blind to experimental status. **(C)** Surface rendered 3D images were generated from confocal Z-stacks of HDF and used to calculate the mean mitochondrial volume per cell in control transfected and sacsin knockdown cells. **(D)** The number of individual mitochondrion was also quantified in control transfected and sacsin knockdown cells, after 1 hour treatment with vehicle control or 20µM CCCP to induce mitochondrial fission. Statistical significances were determined by T test. Error bars = SD.

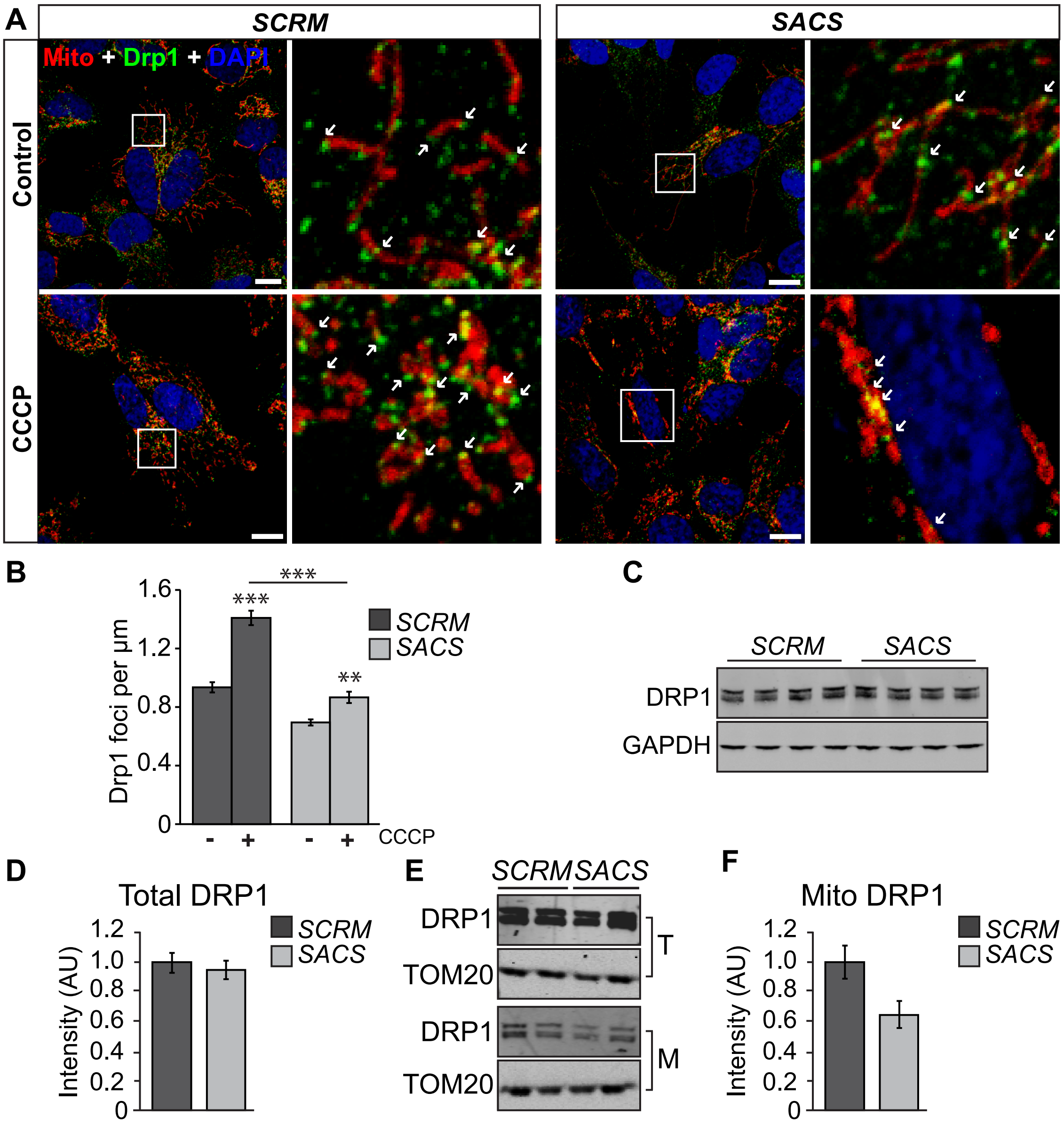

Figure S4. Localisation of Drp1 to mitochondria is reduced in sacsin knockdown SH-SY5Y cells. (A) SH-SY5Ys were cotransfected with control scrambled (SCRM) siRNA or siRNA targeting sacsin (SACS) and DSRed2-mito (red). After 48 hours cells were treated for 1 hour with CCCP and then processed for immunofluorescent detection of Drp1 (green) and counterstained with DAPI (blue) for nuclei. Examples of mitochondrial associated Drp1 foci are indicated by arrows. Scale bar = 10µm. **(B)** The number of Drp1 foci that localised to mitochondria in SCRM and SACS cells with and without CCCP treatment were then quantified from confocal Z-stacks. This data was expressed as the number of Drp1 foci localised to mitochondria per 1 µm of measured length (quantification was performed from at 14 cells per treatment, from three independent replicates). Statistical significances were determined by T test. Error bars = SEM. ** p≤0.001, *** p≤0.005. **(C)** Levels of Drp1 in SCRM and SACS cells were assessed by immunoblot, while GAPDH was used as a loading control. **(D)** Quantitative analyses by densitometry indicated that 48 hours post transfection total cellular levels of Drp1 were not significantly altered in sacsin knockdown cells. **(E)** Representative immunoblot of SH-SY5Y cells transiently transfected with *SACS* or *SCRM* siRNA and subjected to subcellular fractionation. Total (T) and mitochondrial (M) fractions were immunoblotted for Drp1 and TOM20. **(F)** Densitometric analysis of DRP1 levels in the mitochondrial fraction relative to TOM20 suggests a reduction in *SACS* compared to *SCRM* siRNA treated cells (n = 2). Error bars = SD (in D and F)

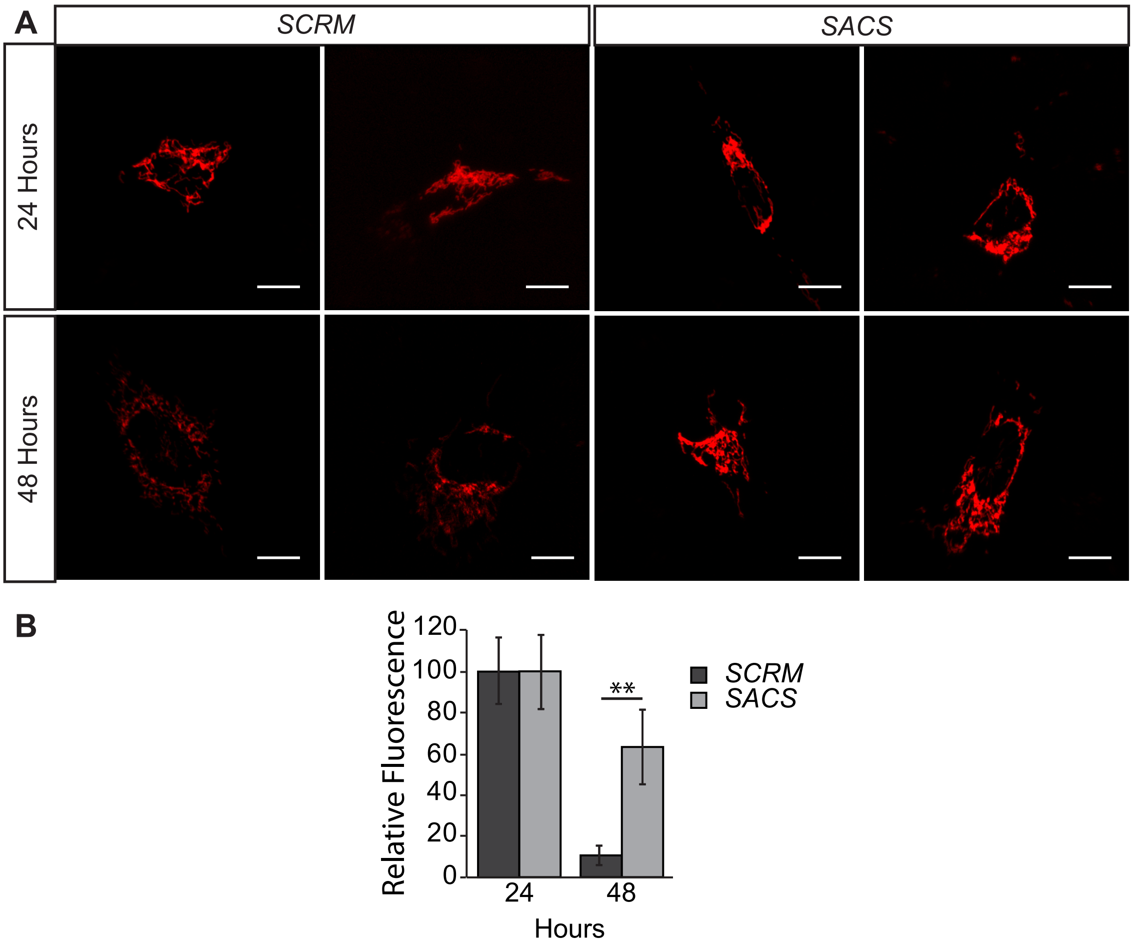

Figure S5. Mitochondrial turnover is decreased in sacsin knockdown cells. (A) Representative images of SH-SY5Y cells 24 and 48 hours after a pulse of MitoTimer protein expression. Tet-On SH-SY5Y were cotransfected with control scrambled (*SCRM*) siRNA or siRNA targeting sacsin (*SACS*) and pTRE-tight-MitoTimer (red). After 24 hours cells were treated for 1 hour with doxycycline and then returned to normal media for a further 24 or 48 hours prior to fixation and confocal imaging. **(B)** MitoTimer red fluorescent intensity was then quantified in individual cells from confocal maximum intensity projections (data is from a representative experiment, where a minimum of 9 cells was imaged per condition). MitoTimer green fluorescence was not at quantifiable levels at 48 hours post the doxacycline treatment in control or sacsin knockdown cells, consistent with a short period of MitoTimer expression, and is not shown. Error bars = SEM. ** $p \leq 0.005$.

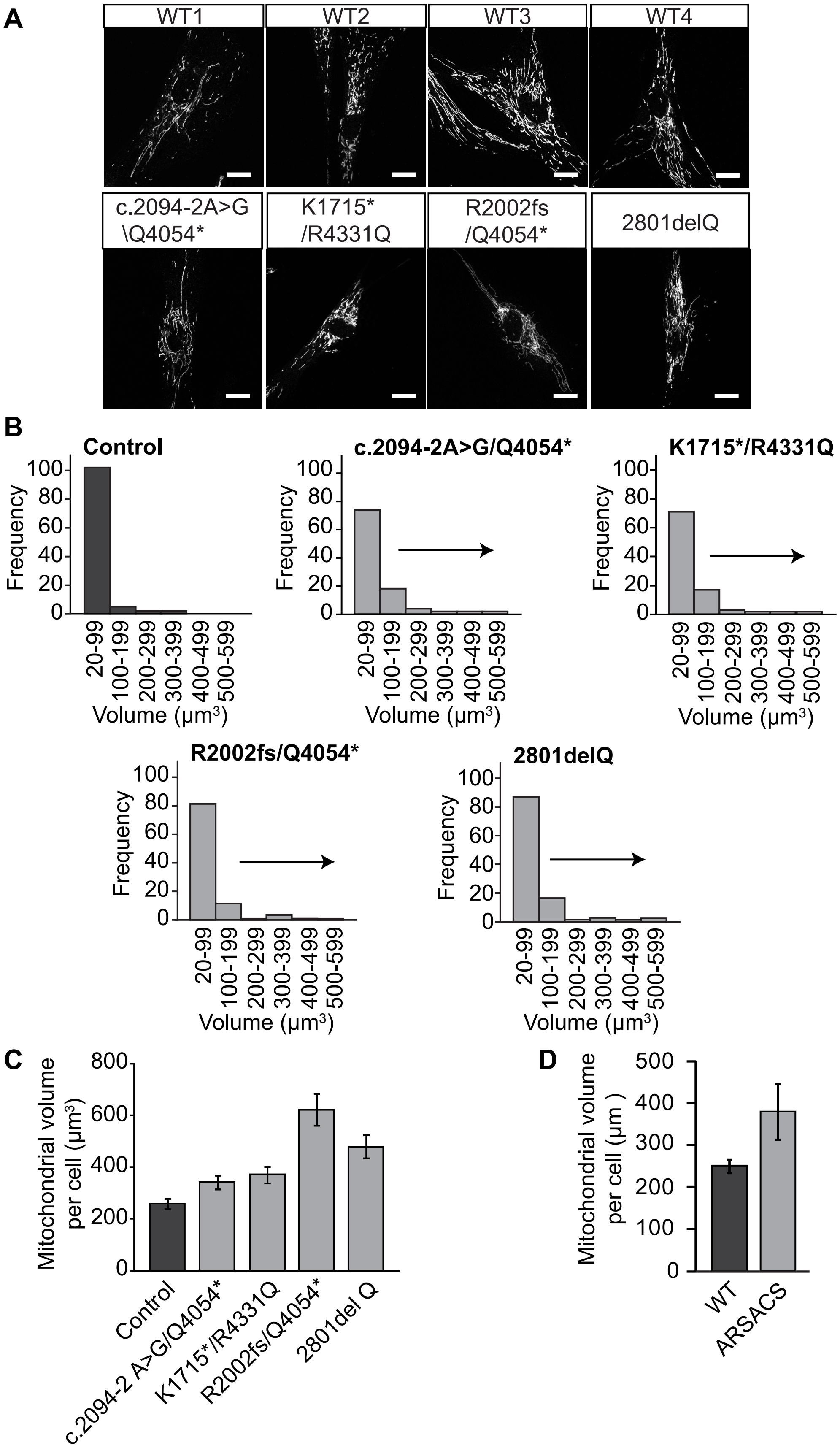

Figure S6. Mitochondrial network organisation is altered in ARSACS patient HDFS. (A) Confocal Z-stacks of live control and patient HDFs stained with MitoTraker were acquired to generate maximum intensity projections. Scale bar = 10μm **(B)** Surface rendered 3D images were generated from this data and used to calculate the number of individual mitochondria and their volume. This is shown as the frequency of individual mitochondria for a representative control cell line and each patient analysed. **(C-D)** From these data the average mitochondrial volume per cell (in μm^3) was calculated. This is shown for each patient cell line (C) and cumulative for the 4 patient cells lines analysed (D). Measurements were made in at least 25 cells per line. Error bars = SEM.

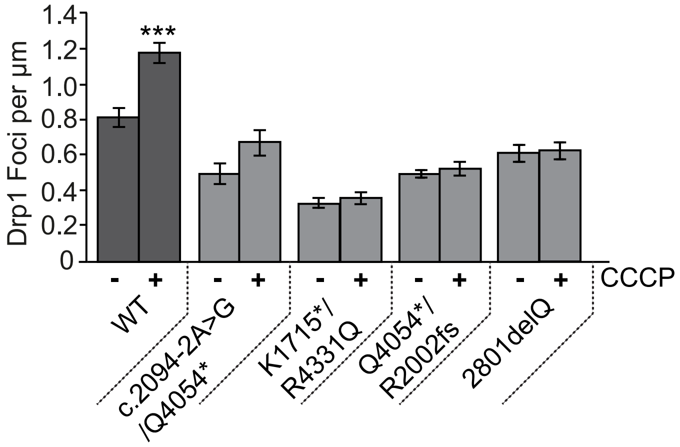

Figure S7. Localisation of Drp1 to mitochondria is reduced in all ARSACS patient cell lines. (A) Control and patient HDFs were treated for 1 hour with CCCP and then processed for immunofluorescent detection of mitochondria and Drp1. The number of Drp1 foci that localized to mitochondria in control and patient HDFs with and without CCCP treatment were then quantified from confocal Z-stacks. This is shown for individual patient cell lines. This data was expressed as the number of Drp1 foci localized to mitochondria per 1 µm of measured length (quantification was performed from at least 6 mitochondria in 45 cells for each treatment, from three independent replicates). Error bars = SEM. *** p≤0.005.
